# Changes in quality of life of early-stage lung cancer patients undergoing sublobar resection: a systematic review

**DOI:** 10.3389/fsurg.2025.1542036

**Published:** 2025-02-28

**Authors:** Dimitrios E. Magouliotis, Ugo Cioffi, Fabrizio Minervini, Savvas Lampridis, Angelo Guttadauro, Marco Scarci

**Affiliations:** ^1^ Department of Cardiac Surgery Research, Lankenau Institute for Medical Research, Wynnewood, PA, United States; ^2^ Department of Surgery, University of Milan, Milan, Italy; ^3^ Division of Thoracic Surgery, Cantonal Hospital Lucerne, Lucerne, Switzerland; ^4^ Department of Thoracic Surgery, 424 General Military Hospital, Thessaloniki, Greece; ^5^ Department of Cardiothoracic Surgery, Hammersmith Hospital, Imperial College Healthcare, National Health Service (NHS) Trust, London, United Kingdom

**Keywords:** quality of life, sublobar lung resection, lobectomy, lung cancer, stereotactic body radiotherapy (SBRT)

## Abstract

**Objective:**

This systematic review aimed to evaluate the impact of sublobar resection (SLR) on the quality of life (QoL) of patients with early-stage non-small cell lung cancer (NSCLC). Specifically, it compared outcomes between sublobar resection, lobectomy, and stereotactic body radiation therapy (SBRT).

**Methods:**

A literature search was conducted across PubMed and Scopus, identifying studies published from 2010 to 2024 that reported QOL outcomes in early-stage NSCLC patients treated with lobectomy, SLR, or SBRT. Inclusion criteria were studies with more than 10 patients, written in English, and using validated QoL metrics. Data on demographics, interventions, QoL tools, and findings were extracted, and study quality was assessed using the Newcastle-Ottawa Scale and the ROBINS-I tool.

**Results:**

Five studies involving 1,149 patients from six countries met the inclusion criteria. QoL outcomes consistently favored SLR over lobectomy in domains such as physical and respiratory function, with SLR patients experiencing faster recovery and fewer complications. Minimally invasive techniques, such as video-assisted thoracoscopic surgery (VATS), further enhanced these outcomes. SBRT demonstrated stable QOL post-treatment but lacked the long-term physical recovery benefits observed with SLR. Commonly employed QoL tools included the EORTC QLQ-C30, Leicester Cough Questionnaire, and NSCLC-PQOL, each capturing distinct dimensions of patient QoL status.

**Conclusion:**

Sublobar resection provides significant QoL benefits for selected early-stage NSCLC patients compared to lobectomy, particularly in respiratory health and recovery endpoints. These findings highlight the value of personalized surgical approaches and the need for further research on optimizing QoL in NSCLC management.

## 1 Introduction

Non-small cell lung cancer (NSCLC) is the most common histologic type of lung cancer, accounting for approximately eighty-five percent of all cases worldwide, thus representing a leading cause of cancer-related mortality due to its high prevalence and often late-stage diagnosis (1). Recent advances in screening techniques and domestic policies (e.g., the introduction of low-dose CT scans and their implementation through statewide screening policies) have increased the diagnosis of early-stage NSCLC, which now accounts for a substantial portion of lung cancer diagnoses (2, 3). In fact, NSCLC represents about 85% of all lung cancer types, with early-stage cases (stage I/II) representing around 20%–30% of the total new diagnoses, especially among individuals who are screened or present with incidental findings (4). The detection of lung cancer in these early stages is critical, as it significantly enhances overall survival, thus highlighting the impact of screening and surveillance efforts in reducing lung cancer mortality (5).

In recent years, the focus of treatment for early-stage NSCLC has expanded beyond survival outcomes to include the quality of life (QoL) of patients undergoing different interventions and treatments. For patients with early-stage NSCLC, surgical resection remains the gold standard, with lobectomy historically recommended based on its superior survival outcomes in tumors greater than 2 cm in size (4, 5). However, as patient-centered care grows in importance, aggressive treatment approaches like sublobar resection (segmentectomy or wedge resection) and stereotactic body radiation therapy (SBRT) have gained increasing popularity due to their potential to preserve lung parenchyma and function, minimize complications, and reduce postoperative morbidity burden (6–9). In particular, sublobar resection, which preserves a larger portion of lung parenchyma compared to conventional lobectomy, has emerged as a feasible alternative for patients with limited respiratory reserve or other significant comorbidities. This shift in focus has led to a growing body of evidence examining not only the oncological effectiveness of these options but also their impact on patient-reported QoL outcomes.

QoL represents a multidimensional construct evaluating physical and emotional status, along with social well-being, thus making it a pivotal tool for assessing the overall success of cancer treatment (10). Regarding patients with NSCLC, the disease might affect QoL in terms of respiratory symptoms, physical limitations, emotional distress, and social isolation, which collectively influence recovery trajectories and long-term patient satisfaction (10, 11). In fact, there is certain evidence demonstrating that lobectomy might lead to substantial postoperative respiratory morbidity, fatigue, and psychological distress due to its more aggressive nature compared to sublobar resection (10–12). In contrast, sublobar resection and SBRT offer lung-sparing benefits that may mitigate these adverse outcomes and facilitate an enhanced recovery pathway (12). Taking everything into consideration, comparative studies evaluating QoL outcomes in sublobar resection vs. lobectomy or SBRT are essential to guide treatment choices for early-stage NSCLC. The aim of this systematic review was to summarize these studies to provide insight into how different surgical and non-surgical interventions influence patients’ day-to-day lives and to highlight the importance of personalized approaches that prioritize both survival and quality of life.

## 2 Methods

### 2.1 Search and articles selection strategy

The current review was designed in accordance with the protocol agreed upon by all authors and the Preferred Reporting Items for Systematic Reviews and Meta-Analyses (13). A systematic literature search was performed in two databases: (1) Pubmed (Medline), and (2) Scopus (ELSEVIER) (last date of literature search: November 10th, 2024). The following terms were used in all possible combinations: “lung cancer”, “non-small cell lung cancer”, “NSCLC”, “lung resection”, “pulmonary resection”, “lobectomy”, “segmentectomy”, “sublobar resection”, “quality of life”, “qol”. Inclusion criteria were (1) original reports with > 10 patients, (2) published from 2010 to 2024, (3) written in English, (4) conducted on human subjects, and (5) reporting outcomes on QoL metrics of patients with NSCLC undergoing lobectomy or sublobar resection or SBRT. We chose to implement a strict time period limit for the inclusion of articles to reduce a potential heterogeneity bias regarding the treatment protocols. We excluded all duplicate articles and hand-searched the reference lists of all articles that were included for additional studies. Two independent reviewers (DEM, SL) extracted data from the included studies. Any potential discrepancies between the two investigators regarding the inclusion/exclusion of the selected studies were discussed with a senior author (MS) to incorporate only the articles that best matched the criteria until a consensus was reached.

### 2.2 Data extraction and quality assessment

For every included study, we extracted data relative to the population, country, study design, intervention, QoL metrics employed, and key findings. To evaluate the quality appropriateness of the included non-RCTs we employed the Newcastle-Ottawa Scale (NOS) (14). The scale uses a range varying from 0 to 9 stars, and studies with a score equal to or higher than five stars were considered to have adequate methodological quality. In addition, the included studies were systematically assessed for risk of bias by employing the Risk of Bias in Non-Randomized Studies of Interventions tool (ROBINS-I) (15). Two reviewers (DEM, SL) rated the studies independently and discrepancies were discussed until a consensus was reached.

## 3 Results

### 3.1 Patient demographics and operative characteristics

**Figure 1:**
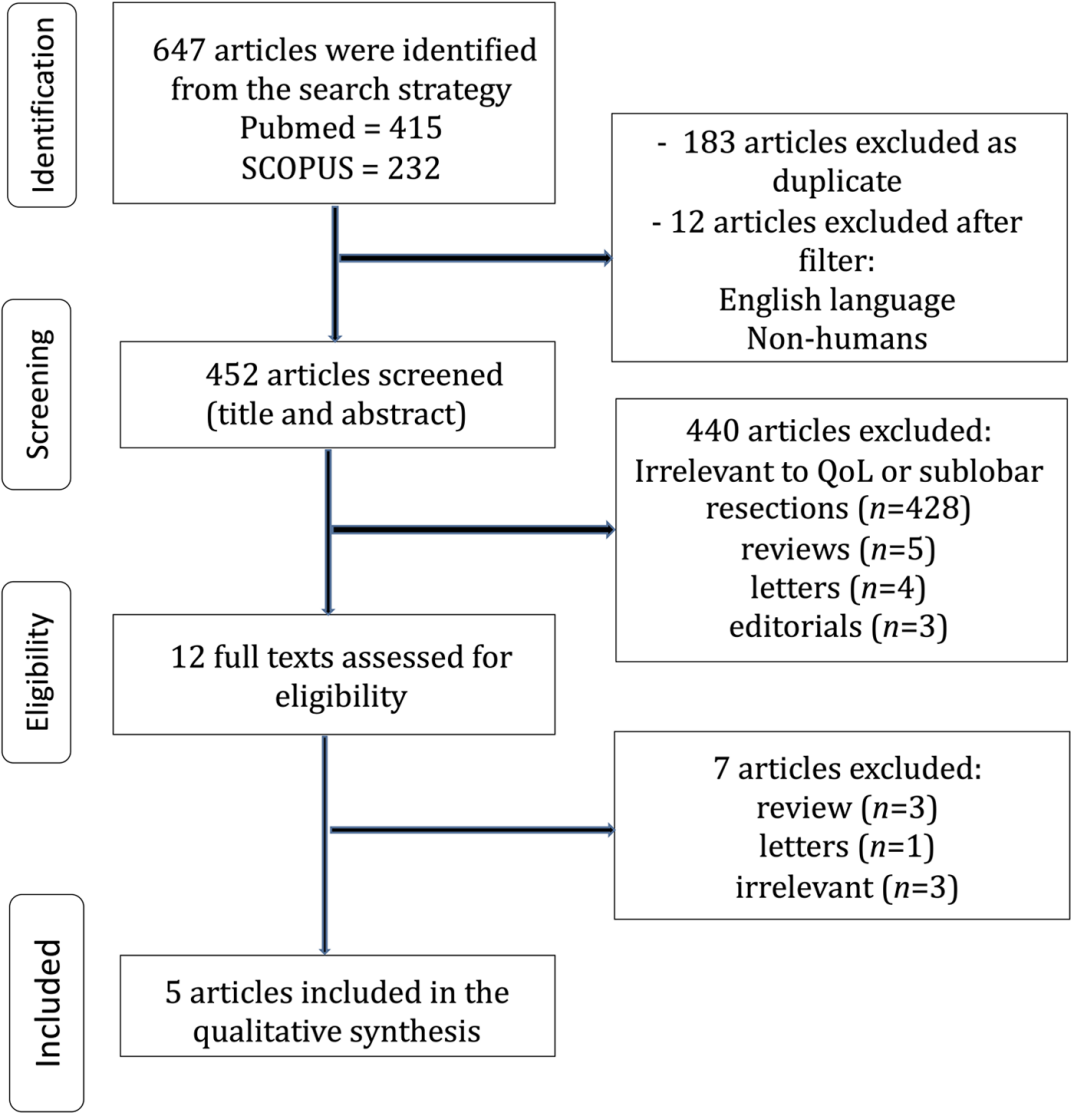
Trial flow of the systemaric review.

**Figure 2:**
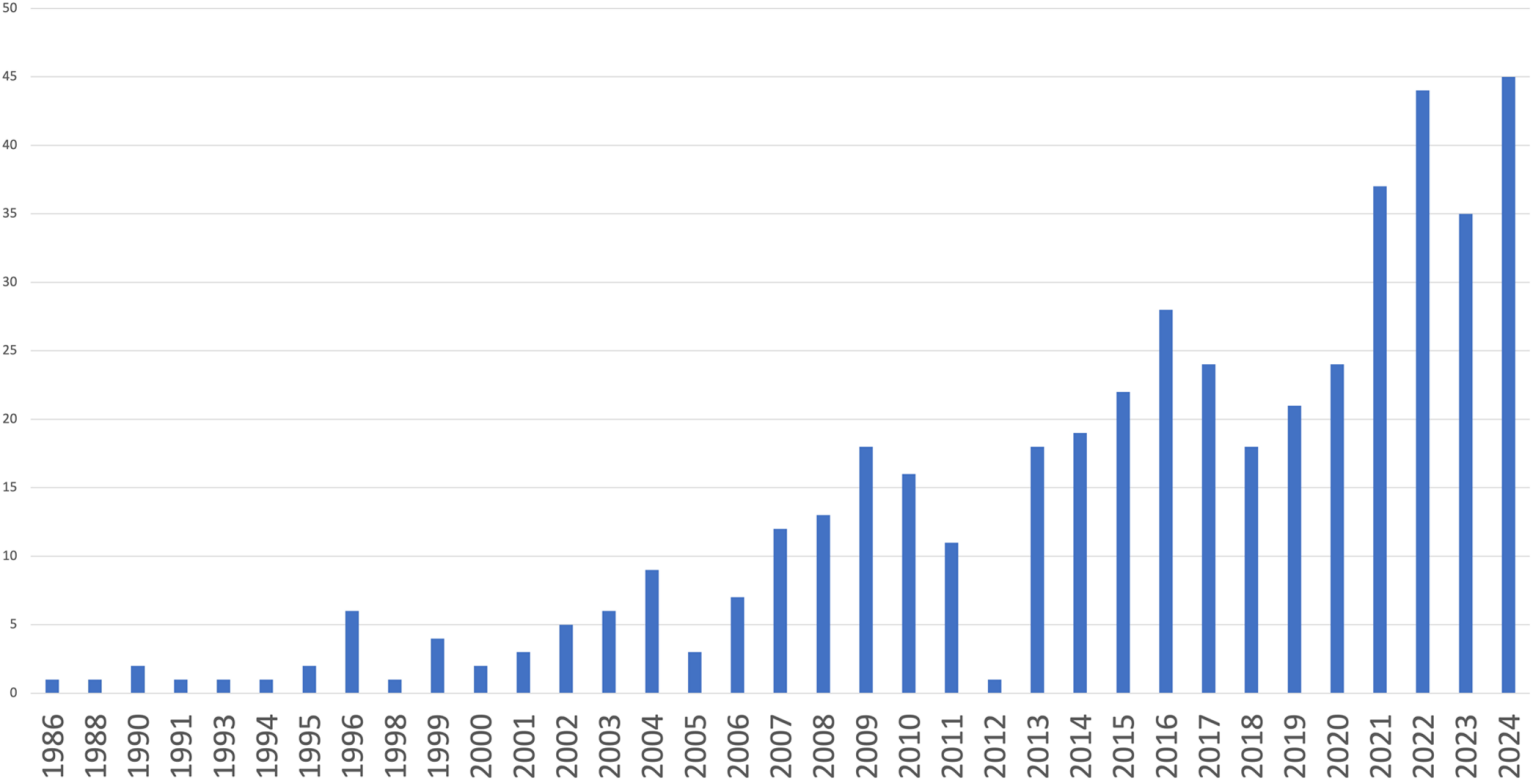
Published articles on the topic of quality of life for patients with early-stage NSCLC undergoing surgery.

**Figure 3:**
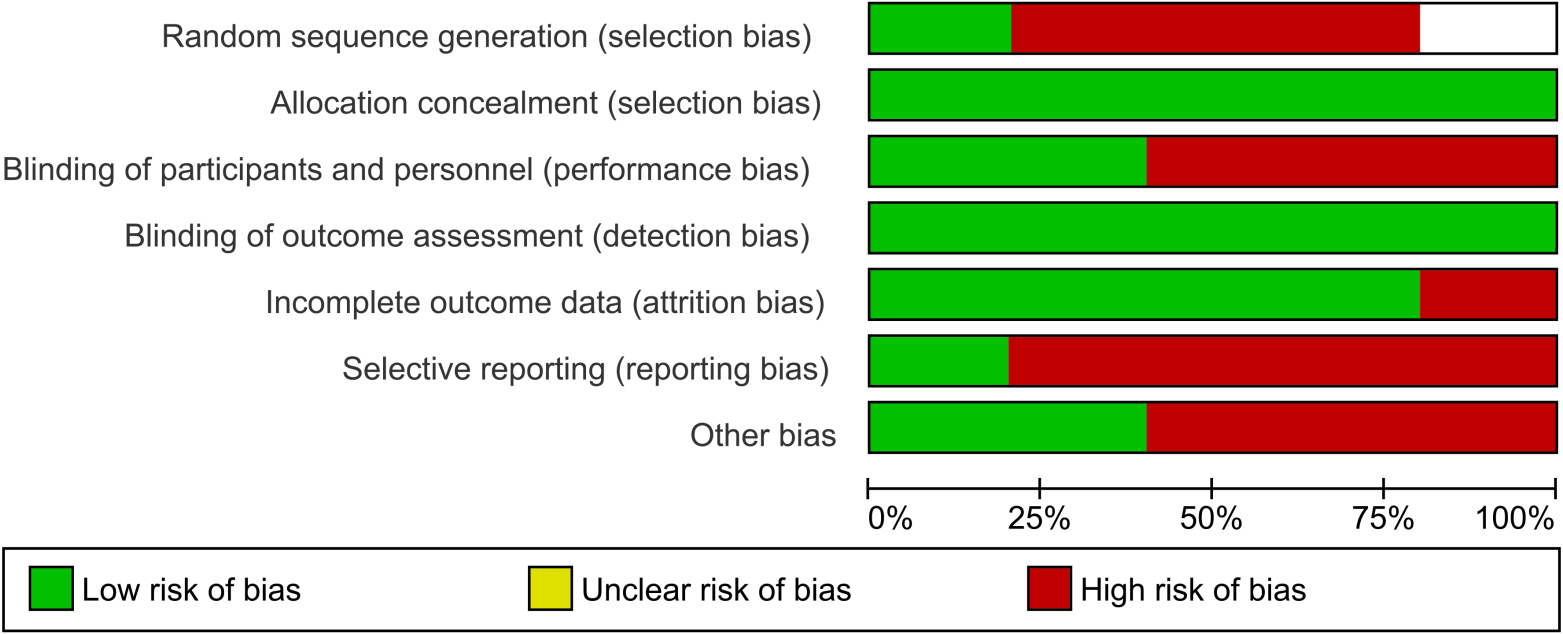
Risk of bias graph.

**Figure 4:**
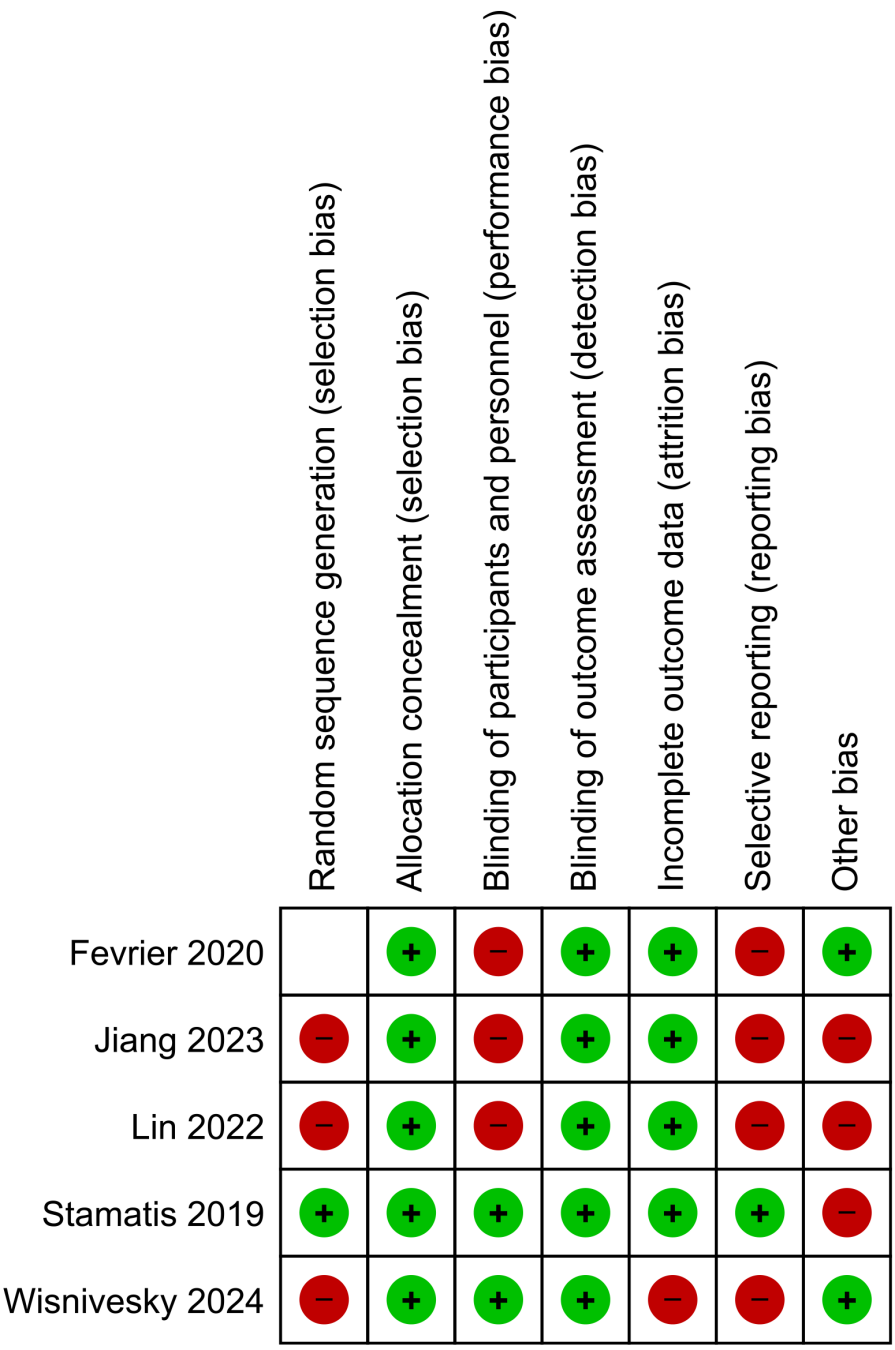
Risk of bias summary.

The flow diagram regarding the search strategy is provided in Figure 1 and the Prisma Checklist is demonstrated in Supplementary Table S1. The characteristics of the incorporated studies are demonstrated in Table 1. Over the last three decades, there has been a great increase in published articles on the topic of QoL for patients with early-stage NSCLC undergoing surgical intervention as demonstrated in Figure 2. From the articles that were retrieved originally, five studies (16–20) were finally included in the present review. The level of agreement between the reviewers was “almost perfect” (kappa = 0.833; 95% CI: 0.604, 1.000). All studies were retrospective and implemented data from nationwide databases. The included studies were published between 2019 and 2024. The present review incorporated data from a total of 1,149 patients from China, France, the United States, Germany, Switzerland, and Austria. The assessment of quality and existence of potential bias using the Newcastle-Ottawa Scale and the ROBINS-I tool are demonstrated in Table 1 and Figures 3, 4.

**Table 1:** Characteristics of the studies and patients that were included in the present review.

Study ID	Study DESIGN	Country	Population	Intervention	QoL metrics	NOS	Key findings
Février et al., (16)	P	France	201 stage IA NSCLC patients treated with VATS SLR or L	SLR vs. L	SF-12, FACT-LCS, PHQ-4	8	SLR patients experienced faster recovery and lower decline in physical health scores, with improved QoL outcomes at one year compared to L patients.
Jiang et al., (17)	R	China	347 NSCLC patients undergoing VATS SLR or L	SLR vs. L	NSCLC-PQOL (customized scale)	7	SLR patients had better respiratory outcomes (e.g., chest tightness, breathlessness) and recovered baseline QoL faster within six months compared to L.
Lin et al., (18)	L	China	156 early-stage NSCLC patients with postoperative cough, treated with VATS SLR or L	SLR vs. L	Leicester Cough Questionnaire (LCQ-MC)	7	VATS SLR patients reported significantly faster recovery in cough-related QoL, particularly in physical and social aspects, compared to L patients. Overall, SLR showed better cough recovery.
Stamatis et al., (19)	RCT	Germany, Switzerland, Austria	108 patients with stage IA NSCLC undergoing either SLR or L	SLR vs. L	EORTC QLQ-C30 and LC13	9	SLR led to significantly better recovery in physical, cognitive, and social functioning up to 12 months postoperatively, whereas L patients had lingering decline in physical and cognitive QoL.
Wisnivesky et al., (20)	P	USA	337 stage I–IIA NSCLC patients at high risk for lobectomy undergoing SBRT or SLR	SLR vs. SBRT	SF-8, FACT-L	8	SBRT patients showed better immediate post-treatment QoL, but by 12 months, both treatment groups returned to baseline QoL levels, indicating similar long-term impact.

SLR, sublobar resection; L, lobectomy; SBRT, stereotactic body radiation therapy; NOS, Newcastle-Ottawa Scale; RCT, randomized controlled trial; P, prospective; L, longitudinal; R, retrospective.

### 3.2 Understanding quality of life in early-stage NSCLC

Quality of life represents a multidimensional concept encompassing physical, psychological, and social parameters, through which it reflects the overall well-being of patients during the perioperative pathway. In the context of treating NSCLC, QoL outcomes are particularly important given the significant morbidity associated with the disease itself and the treatment. Postoperative complications such as dyspnea, pain, fatigue, and reduced physical functioning are common and can profoundly affect the quality of daily life. Moreover, psychological stressors like anxiety, depression, or fear of recurrence add additional layers of complexity to the assessment of QoL perceived by patients.

Sublobar resection is believed to mitigate some of these challenges by preserving more lung parenchyma than lobectomy, potentially reducing postoperative respiratory symptoms and enabling quicker recovery (21, 22). Additionally, the adoption of minimally invasive surgical techniques, such as video-assisted thoracoscopic surgery (VATS), has further reduced the perceived pain, the length of hospital stay, and the cosmetic outcome, thus contributing to enhanced QOL outcomes compared to traditional open thoracotomy (22, 23). These advantages make sublobar resection a favorable treatment option for patients with early-stage NSCLC, especially when considering the growing emphasis on patient-centered care and survivorship quality.

### 3.3 Comparison of surgical approaches and QoL outcomes

The choice between sublobar resection and lobectomy for early-stage NSCLC has been a topic of significant debate in the literature, particularly in the context of balancing oncologic efficacy with QoL outcomes. Lobectomy, the mainstay procedure that involves the excision of the entire lobe of the lung, has traditionally been the gold standard for resectable NSCLC due to its potentially superior survival outcomes. Nonetheless, as more evidence demonstrated the non-inferiority of sublobar resections in terms of oncologic outcomes, the focus was shifted towards the differences in terms of QoL (21). In fact, the more aggressive nature of lobectomy often results in a significant postoperative decline in physical functioning, clinical status, respiratory capacity, and overall QoL, especially in patients with pre-existing comorbidities or limited pulmonary reserve (22). Sublobar resection, including wedge resection and segmentectomy, offers a lung-sparing alternative that has been shown to preserve pulmonary function and expedite recovery, making it an increasingly viable option for select patients.

Studies consistently highlight the QoL benefits of sublobar resection compared to lobectomy, particularly in terms of physical and respiratory health (22). According to Février et al. (16) the sublobar resection was associated with milder clinical decline as demonstrated in terms of physical health scores during the early postoperative period. Patients undergoing sublobar resection recovered faster, with a significant number returning to baseline physical functioning and reporting better QoL outcomes at one year postoperatively compared to their lobectomy counterparts. Key dimensions, such as physical activity levels and the ability to perform daily tasks, showed greater and faster improvement in the sublobar group (16). Jiang et al. (17) similarly reported that patients undergoing sublobar resection experienced a lower incidence of postoperative respiratory complications during the early recovery phase, thus allowing the earlier return to normal activities in a period of six months​ (17).

Perhaps, the most prominent advantage of sublobar resection is the preservation of lung parenchyma and function, which directly correlates with respiratory-related QoL outcomes. By resecting less parenchyma, sublobar resection minimizes the reduction in terms of forced expiratory volume (FEV_1_) during spirometry and diffusion capacity of the lungs for carbon monoxide (DLCO), important metrics of respiratory clinical status (10). In this context, Lin et al. (18) demonstrated that patients undergoing sublobar resection reported significantly less impairment in respiratory-related QoL metrics, including physical, emotional, and social domains, compared to patients undergoing lobectomy. This is particularly relevant for patients with borderline pulmonary function, characterized by reduced respiratory capacity following lobectomy which can lead to chronic dyspnea, fatigue, and a diminished ability to engage in physical activities​ (18).

In terms of mental health, sublobar resection has also been associated with certain advantages over lobectomy. There is certain evidence suggesting that the less invasive nature of the procedure, combined with faster recovery and lower incidence of postoperative morbidity, leads to lower levels of anxiety and depression in the postoperative period (19). In fact, Stamatis et al. (19) reported that segmentectomy patients demonstrated superior emotional functioning scores compared to lobectomy patients at six and twelve months postoperatively. These findings suggest that sublobar resection-associated improved clinical status and faster return to normal activities contribute to enhanced mental health outcomes, overall experience, and QoL (19).

Minimally invasive techniques, such as video-assisted thoracoscopic surgery (VATS) and robotic-assisted thoracoscopic surgery (RATS), have further augmented the QoL benefits of sublobar resection (22). These approaches reduce the surgical trauma associated with open thoracotomy, resulting in shorter hospital stays, lower levels of perceived postoperative pain, and faster recovery times. For instance, in their systematic review Iovoli et al. (22) highlighted that VATS sublobar resection patients consistently reported lower pain scores and faster recovery of physical and social functioning compared to those undergoing open lobectomy. The adoption of minimally invasive techniques also makes sublobar resection accessible to a broader range of patients, including those who might not otherwise be considered suitable candidates for surgery (22).

Despite the enhanced QoL short- and mid-term outcomes of the sublobar resection, it is important to note that not all patients are suitable candidates for this approach. In fact, certain tumor characteristics, such as size, location, and histological subtype, along with the presence of nodal involvement, play a critical role in determining the most appropriate surgical strategy. On the other hand and regarding more extensive tumors, lobectomy remains the preferred approach due to its ability to achieve broader surgical margins and adequate lymph node sampling evaluation. Nonetheless, for patients with smaller, peripheral tumors (≤2 cm) and limited nodal involvement, sublobar resection provides a compelling alternative that prioritizes lung preservation and QOL without compromising oncologic outcomes (22). For patients with early-stage NSCLC, especially those with comorbid conditions or limited pulmonary reserve, the superior QoL outcomes associated with sublobar resection make it an attractive option. However, maintaining a balance between oncologic efficacy and QoL requires careful preoperative assessment, precise surgical technique, and ongoing patient monitoring.

### 3.4 Sublobar resection vs. SBRT

For patients unable to undergo surgery, stereotactic body radiation therapy (SBRT) offers a non-invasive alternative with comparable oncologic outcomes for stage I NSCLC. Certain studies (20) such suggest that SBRT patients maintain stable QoL without the immediate postoperative declines seen in surgical cohorts. However, sublobar resection demonstrates advantages in long-term QoL recovery, particularly in physical domains, highlighting a trade-off between the acute and chronic impacts of these treatments (20).

### 3.5 Quality of life tools used in the included studies

The importance of measuring QoL as a critical component of assessing treatment outcomes in patients with early-stage NSCLC, highlights the pivotal role of employing well validated tools. The studies included in this review utilized several standardized QoL assessment tools to assess the multidimensional effect of surgical intervention on patients’ QoL status. These tools, validated for use in cancer populations, measure a range of physical, emotional, and social domains, providing comprehensive insights into patient postoperative well-being. Below are summarized the QoL tools that were implemented in the included studies.

#### 3.5.1 European organisation for research and treatment of cancer quality of life questionnaire (EORTC QLQ-C30 and QLQ-Lc13)

The EORTC QLQ-C30 (24) is a widely used cancer-specific questionnaire designed to assess QoL in oncology patients. It includes 30 items covering global health status, five functional scales (physical, role, emotional, cognitive, and social), and nine symptom scales regarding fatigue, dyspnea, and pain. The QLQ-LC13 (25) tool is a lung cancer-specific supplement that evaluates additional symptoms such as hemoptysis, chest pain, and side effects of treatment like dysphagia and neuropathy. In their study (19), Stamatis and his team utilized these tools to compare QoL outcomes between segmentectomy and lobectomy patients. Segmentectomy patients reported faster recovery in terms of physical and emotional functioning, with a lower incidence of respiratory morbidity and better overall QoL scores at 12 months postoperatively. In addition, in their article Février et al. (16) employed the QLQ-C30 to measure functional recovery and demonstrated that sublobar resection patients reported enhanced outcomes regarding QoL, along with faster improvement compared to lobectomy patients.

#### 3.5.2 Functional assessment of cancer therapy—lung (FACT-L)

The FACT-L (25) is a validated questionnaire for lung cancer patients, including a total of 36 items divided into the following subscales: physical, social, emotional, functional well-being, and a lung cancer-specific module. It is particularly effective in capturing disease-specific symptoms and treatment-related impacts on QoL. Iovoli et al. (22) included FACT-L in their systematic review, reporting that sublobar resection patients achieved better scores in physical and functional well-being compared to those undergoing more extensive surgical interventions. This tool was instrumental in identifying differences between surgical and non-surgical treatments like SBRT​ (22).

#### 3.5.3 Short form health survey (SF-12 and SF-36)

The SF-12 and SF-36 are generic health-related QoL instruments that assess physical and mental health through component summary scores (26, 27). These tools are often used alongside disease-specific measures to provide a broader perspective on patient well-being. Février et al. (16) employed the SF-12 to evaluate physical and mental health in sublobar resection and lobectomy patients. The study found that sublobar resection patients demonstrated superior physical component scores and recovered faster in both physical and mental health domains (16). Iovoli et al. (22) included SF-36 in their review, highlighting its utility in comparing surgical and non-surgical treatment modalities for early-stage NSCLC.

#### 3.5.4 Leicester cough questionnaire (LCQ)

The Leicester Cough Questionnaire (LCQ) is a well-validated tool designed to assess the impact of chronic cough on QoL. It includes domains such as physical, psychological, and social functioning, making it particularly relevant for NSCLC patients experiencing postoperative cough (28). Lin et al. (18) utilized the LCQ to measure recovery from postoperative cough following sublobar resection and lobectomy. Their findings showed that sublobar resection patients had significantly faster recovery in all domains, emphasizing the QoL benefits of lung-sparing techniques (18).

#### 3.5.5 NSCLC patient-reported quality of life questionnaire (NSCLC-PQOL)

The NSCLC-PQOL is a customized tool designed to evaluate specific symptoms and QoL challenges associated with NSCLC treatment (17). It includes questions focusing on respiratory symptoms, general health, and treatment satisfaction. Jiang et al. (17) employed this tool to assess the QoL outcomes of patients undergoing sublobar resection vs. lobectomy. The study highlighted superior respiratory-related QoL scores for sublobar resection patients, particularly in reducing breathlessness and chest discomfort (17).

## 4 Discussion

The growing body of evidence supporting sublobar resection as an alternative to lobectomy for early-stage non-small cell lung cancer highlights the evolving role of QoL-related considerations affecting the changing landscape of thoracic oncology, alongside traditional oncologic outcomes. Sublobar resection, particularly when performed through a minimally invasive approach, offers several advantages that align with patient-centered care principles, including faster recovery, preservation of pulmonary function, and reduced postoperative morbidity. However, recent findings from large-scale randomized controlled trials (RCTs), such as the Japanese Clinical Oncology Group (JCOG) studies, provide crucial insights that must be taken into account when evaluating the appropriateness of sublobar resection for certain patient populations. The JCOG0802/WJOG4607l trial (21) compared segmentectomy to lobectomy for early-stage NSCLC and revealed an unexpectedly small difference in the reduction of median forced expiratory volume in 1 s (FEV1) at 12 months postoperatively—only 3.5%—which is significantly lower than the traditionally considered clinically relevant threshold of 10%. This finding suggests that while segmentectomy preserves lung parenchyma, its functional advantage may not be as pronounced as initially expected. Given that pulmonary function is a major determinant of postoperative QoL, further research is warranted to assess long-term respiratory function outcomes following segmentectomy. Additionally, while segmentectomy demonstrated oncologic non-inferiority in terms of overall survival, the study found a higher local recurrence rate in the segmentectomy group compared to lobectomy. This raises concerns regarding the suitability of segmentectomy for patients without significant comorbidities. Traditionally, lobectomy has been the gold standard treatment for early-stage NSCLC, with sublobar resection primarily reserved for high-risk patients or those with limited pulmonary reserve. The findings of increased local recurrence with segmentectomy emphasize the need for careful patient selection when considering intentional sublobar resection for otherwise fit individuals.

The utilization of validated QoL metrics, such as EORTC QLQ-C30 and NSCLC-PQOL, highlights the growing recognition of patient-reported outcomes in thoracic oncology. These tools, widely accepted in clinical research, provide a multidimensional perspective on physical, emotional, and social well-being. Their application facilitates the assessment of treatment impact beyond traditional survival metrics, making them indispensable for evaluating modern surgical and non-surgical interventions. However, the heterogeneity in QoL tools used across studies indicates the need for standardized measures to enable robust comparisons and meta-analyses. The present systematic review included a total of five studies in an effort to dissect the impact of sublobar resection on patients’ quality of life.

The findings on QoL emphasize the importance of personalizing surgical approaches based on tumor characteristics, patient comorbidities, and individual preferences. Sublobar resection is particularly well-suited for patients with small, peripheral tumors (≤2 cm), those with pre-existing respiratory or cardiac comorbidities, or older and frail patients who may be less tolerant of more extensive surgery. The ability to preserve lung parenchyma while achieving equivalent oncologic outcomes makes sublobar resection an ideal option for these patients. Additionally, the shorter recovery times associated with sublobar resection may enable patients to return to normal activities in a shorter period, thus reducing the psychosocial burden of prolonged convalescence. Furthermore, minimally invasive techniques, like VATS and RATS, have further enhanced the viability of sublobar resection by reducing perioperative morbidity and hospital stays (22). Nonetheless, integrating advanced surgical methods requires robust training programs and the availability of experienced thoracic surgeons to ensure consistent outcomes (22).

QoL outcomes from sublobar resection should also facilitate counseling with patients during the shared decision-making processes. By presenting clear data on the potential benefits and limitations of each treatment option, clinicians can empower patients to make informed choices that align with their personal goals and lifestyle priorities. Incorporating validated QoL tools into routine clinical practice can provide a structured framework for evaluating patient-reported outcomes and tailoring follow-up care to address residual symptoms or concerns.

While sublobar resection offers significant advantages in terms of quality of life outcomes, it presents several challenges that must be addressed to optimize patient care and ensure comparable oncologic efficacy to lobectomy. One of the primary concerns with sublobar resection is achieving adequate surgical margins. Unlike lobectomy, which removes an entire lobe of the lung, sublobar resection involves a more limited resection of lung parenchyma, thus increasing the risk of R1 resection (29). This issue is particularly critical for tumors with aggressive growth patterns or indistinct borders, where achieving a sufficient margin (e.g., at least 2 cm or the diameter of the tumor) can be technically challenging. Failure to achieve R0 resection margins may lead to inferior oncologic outcomes, thus undermining the primary goal of curative surgery.

Another challenge lies in the thoroughness of lymph node sampling. Lobectomy typically involves systematic mediastinal lymphadenectomy, allowing for accurate staging and reducing the risk of occult nodal metastases (30). In contrast, sublobar resection may be associated with less extensive lymph node evaluation, particularly in cases where the procedure is performed minimally invasively or when the surgeon prioritizes parenchymal preservation. Studies have shown that inadequate lymph node sampling can result in under-staging, leading to inappropriate omission of adjuvant therapies and increased recurrence risks (31). As a result, guidelines emphasize the importance of performing adequate lymph node sampling even in sublobar resections to maintain a proper oncologic outcome (32).

Patient selection represents another critical consideration. Sublobar resection is generally recommended for specific subgroups of early-stage NSCLC patients, such as those with smaller tumors (≤2 cm), limited ground-glass opacities, or pure adenocarcinoma *in situ* histology (7). Patients with more aggressive tumor biology or evidence of nodal involvement may benefit more from lobectomy. Moreover, while sublobar resection is often favored for patients with comorbidities or poor pulmonary reserve, these same factors can increase the risk of postoperative complications, making patient optimization and perioperative management crucial (33). Large, multicenter randomized controlled trials, such as CALGB 140503, will be instrumental in defining the long-term efficacy of sublobar approaches across diverse populations (21, 33).

Recent and ongoing large-scale randomized controlled trials (RCTs) such as CALGB 140503 (7) and JCOG0802 (21) have provided compelling evidence on the oncologic equivalence of sublobar resection to lobectomy in selected early-stage NSCLC patients. These studies have also brought attention to critical aspects of QoL outcomes, highlighting that less invasive surgeries can preserve pulmonary function and lead to faster physical recovery. In fact, the CALGB 140503 trial (7) demonstrated that segmentectomy achieves similar overall survival and disease-free survival outcomes as lobectomy for tumors ≤2 cm, while offering the added benefits of reduced postoperative complications and improved recovery times. The JCOG0802 trial (21) further supports these findings, emphasizing that segmentectomy, when performed with meticulous lymph node dissection, does not compromise oncologic safety while potentially improving patient satisfaction and respiratory health. Moreover, sublobar resection should not be regarded as a universal alternative to lobectomy. Although it provides QoL benefits, particularly in preserving pulmonary function and expediting recovery, its role should be carefully weighed against oncologic risks. Future studies should aim to identify subgroups of patients who may truly benefit from segmentectomy without compromising long-term cancer control. Until further evidence clarifies these indications, the decision to pursue intentional segmentectomy in patients without significant comorbidities should be approached with caution. Incorporating these findings into future reviews and analyses will help to solidify the role of sublobar resection in clinical practice. The integration of these robust data sources could also address some limitations of existing evidence, such as the heterogeneity in study designs and QoL tools used. Moreover, the insights gained from these trials can guide clinicians in making more informed, patient-centered decisions and inspire future research to refine surgical techniques and perioperative management strategies.

Addressing these challenges requires a multidisciplinary approach, involving thoracic surgeons, pulmonologists, medical oncologists, and radiologists, to ensure optimal patient selection, perioperative care, and follow-up. Furthermore, robust training programs and the integration of advanced technologies will be essential to overcoming technical limitations and ensuring the widespread adoption of best practices in sublobar resection. By addressing these challenges, sublobar resection can continue to provide a viable, QoL-preserving option for patients with early-stage NSCLC.

This systematic review contributes significantly to the evolving paradigm of personalized care in early-stage NSCLC. By synthesizing data from diverse studies, the review underscores the value of prioritizing patient-reported outcomes like quality of life (QoL) alongside traditional oncologic metrics such as survival and recurrence rates. The findings reaffirm the potential of sublobar resection to meet the dual objectives of oncologic efficacy and QoL preservation. This is particularly relevant in the current era of patient-centered care, where treatment success is not solely defined by survival but also by how well patients can return to their daily lives. For patients with limited pulmonary reserve, important comorbidities, or individual preference for less invasive interventions, sublobar resection offers a tailored solution that balances surgical precision with reduced morbidity. Beyond its clinical implications, the study highlights gaps in existing research, such as the variability in QoL measurement tools and the limited number of large-scale comparative studies. By identifying these gaps, the review sets the stage for future investigations to optimize surgical approaches, validate emerging techniques like robotic-assisted segmentectomy, and integrate advanced technologies for personalized treatment planning. In this context, the review serves as a valuable resource for thoracic surgeons, oncologists, and multidisciplinary care teams, supporting informed decision-making and advancing the field of thoracic oncology. Its emphasis on integrating QoL into treatment evaluations contributes to shaping a future where patient outcomes are measured holistically, encompassing both survival and quality of survivorship.

The limitations of the present review are mainly associated with the limitations of the included studies. The small number of included studies (*n* = 5) limits the generalizability of the findings, and the variability in QoL measurement tools, surgical techniques, and treatment protocols introduces potential heterogeneity. Additionally, differences in follow-up duration across studies may impact the consistency of long-term QoL outcomes reported. Moreover, there was on retrospective study, three prospective and one RCT. Furthermore, the incorporated studies are related to biases related to participants’ selection and performance. In addition, the heterogeneity among institutions regarding the QoL tools that were used, the treatment protocols, the selection criteria, and the perioperative management pose several limitations. On the other hand, the strengths of the present review include the clear literature search and data-extraction protocol, the well-specified inclusion/exclusion criteria, the literature search in three databases, the quality assessment of the included studies, and the detailed presentation of the outcomes. Future research incorporating larger-scale RCTs and standardized QoL assessment methodologies is essential to enhance the reliability and applicability of the current findings.

## 5 Conclusion

The integration of sublobar resection into the treatment paradigm for early-stage NSCLC represents a significant advancement in aligning oncologic and QOL outcomes. By addressing current challenges and exploring future opportunities, clinicians can ensure that treatment strategies remain patient-focused, evidence-based, and adaptable to evolving technologies and therapies. As research continues to elucidate the benefits of sublobar resection, its role as a cornerstone of NSCLC management will likely expand, offering hope for improved survivorship experiences and long-term well-being.

## Data Availability

Publicly available datasets were analyzed in this study. This data can be found here: All data are provided in the text. Original data are provided in the cited articles.
